# Fatal metformin-associated lactic acidosis in a healthy woman after massive overdose

**DOI:** 10.1210/jcemcr/luag109

**Published:** 2026-04-28

**Authors:** Elif Unal Kaya, Mert Ali Kaya

**Affiliations:** Department of Intensive Care Medicine, Ankara Bilkent City Hospital, Ankara 06800, Turkiye; Department of Internal Medicine, Ankara Bilkent City Hospital, Ankara 06800, Turkiye

**Keywords:** metformin-associated lactic acidosis, metformin overdose, hemodialysis, lactic acidosis

## Abstract

Metformin is widely prescribed for type 2 diabetes mellitus and is generally considered safe. However, metformin-associated lactic acidosis (MALA) is a rare but potentially fatal complication. Although typically associated with renal or hepatic dysfunction, intentional overdose can also lead to severe acidosis and high mortality. A 42-year-old woman with no medical history ingested 12 g of metformin in a suicide attempt. She presented with stable vital signs and normal renal and hepatic function. Initial arterial blood gas showed metabolic acidosis (pH 7.21, HCO_3_^−^ 12 mmol/L, lactate 8 mmol/L). Intermittent hemodialysis was initiated, but her acidosis worsened. She developed hypotension requiring norepinephrine and subsequently deteriorated neurologically. Despite 2 hemodialysis sessions, she progressed to cardiac arrest and died. This case demonstrates that massive metformin overdose can overwhelm normal metabolic and clearance mechanisms, resulting in refractory lactic acidosis. Even with early hemodialysis, tissue sequestration of metformin and ongoing mitochondrial dysfunction may sustain lactate overproduction. Massive metformin overdose can be fatal even in healthy individuals. Close monitoring is essential, and prolonged or continuous renal replacement therapies may be warranted when initial dialysis fails to correct severe acidosis.

## Introduction

Metformin is the most widely prescribed oral antihyperglycemic agent for type 2 diabetes mellitus due to its efficacy and favorable safety profile [1]. Despite long-standing clinical use, metformin-associated lactic acidosis (MALA) remains a rare but severe complication, with an estimated incidence below 10 cases per 100 000 patient-years [2, 3]. MALA is characterized by metabolic acidosis with elevated lactate concentrations, commonly defined as lactate >5 mmol/L and pH <7.35 in the presence of metformin exposure [4].

The condition usually arises in patients with predisposing factors such as renal impairment, hepatic dysfunction, cardiovascular collapse, or concurrent critical illness [5]. However, acute intentional overdose can also precipitate profound lactic acidosis in patients without underlying disease [6, 7]. Metformin's inhibition of mitochondrial complex I contributes to impaired oxidative phosphorylation and enhanced anaerobic metabolism, promoting lactate accumulation [8, 9].

Although early hemodialysis is considered the cornerstone of management due to metformin's dialyzable properties, clinical outcomes remain variable, particularly after massive overdose [10]. Mortality in such cases can be high, even when baseline renal and hepatic function is normal [11]. We describe a fatal case of MALA in an otherwise healthy woman following ingestion of a very large dose of metformin.

## Case presentation

A 42-year-old woman with no prior medical history presented to the emergency department one hour after ingesting 12 g of metformin, which belonged to her mother as a diabetic patient, in a suicide attempt. She was alert with a Glasgow Coma Scale (GCS) score of 15 and hemodynamically stable (heart rate 72 bpm, blood pressure 116/82 mmHg). She had a BMI of 30.86 kg/m^2^. Physical examination was unremarkable. She had no prior history of suicide attempts.

## Diagnostic assessment

Initial laboratory investigations showed normal hematologic, renal, and hepatic parameters: hemoglobin 13.2 g/dL (SI: 132 g/L) (reference range, 11.9-14.6 mg/dL [SI: 119-146 g/L]), platelets 246 × 10^9^/L (188-358 × 10^9^/L), white blood cells 7.2 × 10^9^/L (reference range, 4.0-10.0 × 10^9^/L), creatinine 0.92 mg/dL (SI: 81 µmol/L) (reference range, 0.5-1.1 mg/dL [SI: 44-97 µmol/L]), estimated glomerular filtration rate 96 mL/min/1.73 m^2^, urea 47 mg/dL (SI: 16.7 mmol/L) (reference range, 19-49 mg/dL [SI: 6.8-17.5 mmol/L]), albumin 38 g/L (reference range, 32-48 g/L), alanine transaminase (ALT) 24 U/L (<50 U/L), aspartate transaminase (AST) 32 U/L (<35 U/L), alkaline phosphatase (ALP) 45 U/L (53-141 U/L), gamma-glutamyltransferase (GGT) 23 U/L (<38 U/L), total bilirubin 1.0 mg/dL (SI: 17.1 µmol/L) (reference range, 0.2-1.1 mg/dL [SI: 3.4-18.8 µmol/L]) and direct bilirubin 0.6 mg/dL (SI: 10.3 µmol/L) (reference range <0.3 mg/dL [SI: <5.1 µmol/L]). Her initial blood glucose level was 82 mg/dL (SI: 4.6 mmol/L) (reference range, 70-99 mg/dL [SI: 3.9-5.5 mmol/L]). Her thyroid stimulations hormone (TSH) level was 2.62 mIU/L (reference range, 0.4-4.5 mIU/L), her free T3 level was 4.5 pmol/L (reference range, 3.1-6.8 pmol/L), and her free T4 level was 15 pmol/L (reference range, 12-22 pmol/L). Her C-reactive protein was <5 mg/L (reference range, <5 mg/L). All results were within normal limits except for a mildly elevated direct bilirubin and low ALP. Her urinalysis was unremarkable. Toxicology screen, which screened for cannabinoids, opioids, cocaine, amphetamines, benzodiazepines, barbiturates, and phencyclidine, showed no signs of these substances. Arterial blood gas showed metabolic acidosis: pH 7.21 (7.35-7.45), HCO_3_^−^ 12 mmol/L (22-28 mmol/L), and lactate 8 mmol/L (0.5-2.0 mmol/L).

Given the history of large metformin ingestion and absence of alternative causes, a diagnosis of early metformin-associated lactic acidosis was made. No evidence of co-ingestion, infection, or organ dysfunction was identified. Imaging was not required.

## Treatment

Intermittent hemodialysis (HD) without ultrafiltration was initiated 8 hours after presentation to enhance metformin clearance and correct acidosis. The first HD session lasted 2 hours. Post-dialysis arterial blood gas demonstrated worsening metabolic derangements with blood gas result showing pH 7.16, HCO_3_^−^ 8 mmol/L, lactate 16 mmol/L. Following this session, the patient became hypotensive (blood pressure 86/42 mmHg) and required norepinephrine infusion at 0.05 µg/kg/min. She had continuous dextrose infusion to target blood glucose levels above 100 mg/dL.

On the following day, at the 28th hour mark, a second HD session lasting 3 hours was performed. Despite treatment, the patient remained profoundly acidotic post-dialysis with blood gas analysis showing pH 7.18, HCO_3_^−^ 10 mmol/L, and lactate 18 mmol/L. The persistent acidosis suggested ongoing mitochondrial dysfunction and inadequate clearance of tissue-sequestered metformin.

## Outcome and follow-up

After the second HD session, the patient's neurological status deteriorated, with GCS declining to 10. Her hemodynamic instability worsened despite vasopressor support. She subsequently developed cardiac arrest, and resuscitation efforts were unsuccessful. Table 1

**Table 1:** Time course of the patient's clinical status

Timepoint	GCS	Vitals	ABG results	Interventions	Notes
Admission (1 hour post-ingestion)	15	HR 72, BP 116/82	pH 7.21, HCO_3_^−^ 12 mmol/L, Lactate 8 mmol/L	Preparation for hemodialysis	Normal CBC, LFT, RFT, electrolytes; toxicology negative
After 1st HD (2 hours, at 8th hour)	15	BP ↓ 86/42 (norepinephrine 0.05 µg/kg/min)	pH 7.16, HCO_3_^−^ 8 mmol/L, Lactate 16 mmol/L	Intermittent HD (2 hours, no ultrafiltration)	Hemodynamic instability developed
After 2nd HD (3 hours, next day at 28th hour)	10	Hypotension, norepinephrine ongoing	pH 7.18, HCO_3_^−^ 10 mmol/L, Lactate 18 mmol/L	Intermittent HD (3 hours, no ultrafiltration)	Neurological deterioration
Outcome (32nd hour)	—	Cardiac arrest	—	—	Died due to refractory lactic acidosis

Abbreviations: µg, microgram; BP, blood pressure; CBC, complete blood count; HD, hemodialysis; HR, heart rate; kg, kilogram; LFT, liver function tests; min, minute; RFT, renal function tests.

## Discussion

MALA is an uncommon but life-threatening condition, typically associated with renal insufficiency, hepatic dysfunction, or systemic hypoperfusion [5, 6]. Acute overdose represents a less common but clinically important pathway to severe toxicity. This case highlights the potential for massive metformin ingestion to cause rapidly progressive lactic acidosis even when baseline renal and hepatic function are normal.

**Figure 1:**
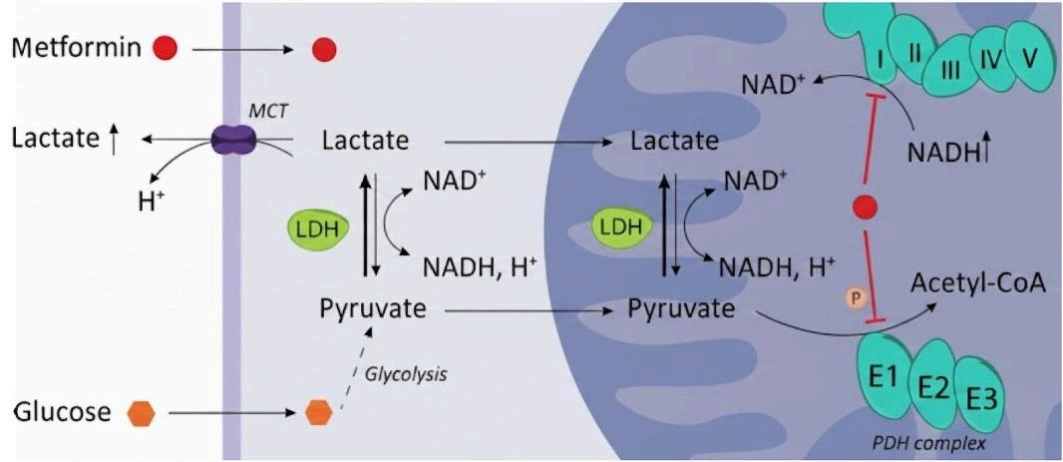
Metformin elevates lactate secretion by forcing the LDH reaction toward lactate production, a process fueled by increased glycolysis. By inhibiting respiratory chain complex I, the drug alters the cellular redox state and promotes the phosphorylation of the PDH complex. This inhibition of pyruvate oxidation significantly contributes to the observed accumulation of lactate [9]. MCT: monocarboxylate transporters, NAD^+^: nicotinamide adenine dinucleotide, NADH: nicotinamide adenine dinucleotide-hydrogen, H^+^: hydrogen.

Despite early recognition and timely initiation of intermittent HD, the patient's acidosis worsened, with rising lactate and increasing vasopressor requirements. Metformin is known to accumulate both in plasma and within tissues, including hepatocytes and skeletal muscle, where it inhibits mitochondrial oxidative phosphorylation and promotes anaerobic lactate production [7, 9]. Tissue sequestration may reduce the efficacy of intermittent HD alone. According to the prognostic and therapeutic analysis by Seidowsky et al, metformin follows a bicompartmental kinetic model, signifying significant tissue sequestration beyond the intravascular space. Their findings highlight that because the drug is stored in peripheral tissues, a prolonged cumulative hemodialysis duration, averaging 15 hours, is typically required to effectively clear these sequestered stores and return plasma concentrations to therapeutic levels. This kinetic behavior underscores the importance of extended renal replacement therapy to prevent the “rebound” of plasma levels from peripheral compartments [10]. Figure 1

Several clinical features in this case—pH <7.1, lactate >15 mmol/L, vasopressor requirement, and neurological decline—have been associated with high mortality in MALA [2, 6, 11]. The persistence of severe acidosis despite 2 HD sessions suggests that continuous renal replacement therapy (CRRT) may be more effective in similar cases. Prior work supports the potential benefits of prolonged or continuous modalities for improving lactate clearance and metabolic stability in refractory MALA [10].

This case reinforces that early clinical stability following massive ingestion may be misleading; rapid deterioration can occur despite guideline-based management. Intensive monitoring and early consideration of continuous extracorporeal therapy may improve outcomes.

Massive metformin overdose can result in rapidly progressive and fatal lactic acidosis even in individuals without underlying renal or hepatic impairment. Deterioration may occur despite early hemodialysis, and clinicians should consider prolonged or continuous renal replacement modalities in severe or refractory cases.

## Learning points

- Massive metformin ingestion can cause rapidly progressive and fatal lactic acidosis even in patients without renal or hepatic impairment.
- Initial clinical stability may be misleading; patients can deteriorate despite early recognition and prompt initiation of hemodialysis.
- Intermittent hemodialysis may be insufficient in severe cases, and prolonged or continuous renal replacement therapies should be considered.

## Contributors

All authors made individual contributions to the authorship. E.U.K and M.A.K were involved in the diagnosis and management of the patient and manuscript submission. All authors reviewed and approved the final draft.

## Data Availability

Data sharing is not applicable to this article as no datasets were generated and analyzed for this article.
